# An Acute Kidney Injury Prediction Model for 24-hour Ultramarathon Runners

**DOI:** 10.2478/hukin-2022-0070

**Published:** 2022-11-08

**Authors:** Po-Ya Hsu, Yi-Chung Hsu, Hsin-Li Liu, Wei Fong Kao, Kuan-Yu Lin

**Affiliations:** 1 Department of Computer Science and Engineering, University of California San Diego, La Jolla, California, the United States.; 2 Department of Nurse Practitioner, Fu Jen Catholic University Hospital, Taipei, Taiwan.; 3 Department of Nursing, Central Taiwan University of Science and Technology, Taichung City, Taiwan.; 4 Department of Emergency Medicine, Taipei Medical University, Taipei, Taiwan.

**Keywords:** acute kidney injury, extreme sports, injury prevention, machine learning

## Abstract

Acute kidney injury (AKI) is frequently seen in ultrarunners, and in this study, an AKI prediction model for 24-hour ultrarunners was built based on the runner’s prerace blood, urine, and body composition data. Twenty-two ultrarunners participated in the study. The risk of acquiring AKI was evaluated by a support vector machine (SVM) model, which is a statistical model commonly used for classification tasks. The inputs of the SVM model were the data collected 1 hour before the race, and the output of the SVM model was the decision of acquiring AKI. Our best AKI prediction model achieved accuracy of 96% in training and 90% in cross-validation tests. In addition, the sensitivity and specificity of the model were 90% and 100%, respectively. In accordance with the AKI prediction model components, ultra-runners are suggested to have high muscle mass and undergo regular ultra-endurance sports training to reduce the risk of acquiring AKI after participating in a 24-hour ultramarathon.

## Introduction

An ultramarathon is a footrace that spans a distance greater than 42.195 km. The participants of ultramarathons, called ultrarunners, are susceptible to dehydration, exhaustion, heat illness, muscle cramps, and rhabdomyolysis (Cheuvront and Haymes, 2001). Severe dehydration can lead to the sudden failure of renal function, which is more well-known as acute kidney injury (AKI) (Powers, 1970).

AKI is frequently observed among ultrarunners, with an incidence rate ranging from 30 to 80% (Lipman et al., 2014). A considerable number of ultrarunners may require hospitalization due to AKI after a race (Bruso et al., 2010; Seedat et al., 1989). The most common etiology of AKI in ultrarunners is rhabdomyolysis, which is caused by muscle breakdown arising from excessively continuous exertion (Hoffman and Weiss, 2016). In light of the severity and the incidence rate of AKI among ultrarunners, an AKI prediction model for ultra-runners during the prerace stage is highly necessary.

Artificial intelligent models have been recently applied in the field of medicine, such as in biomarker identification, physiological status estimation, and medical diagnosis (Chang et al., 2019; Kononenko, 2001). Among various machine learning (ML) approaches, the support vector machine (SVM) is a widely known algorithm that is capable of addressing classification challenges associated with multivariate data (Cortes and Vapnik, 1995). In the present study, ultrarunners’ body composition, urine, and blood data were utilized together with the SVM method to devise an AKI risk evaluation model. Such strategy has been shown in the Hsu et al.’s (2020) study to successfully predict the AKI incidence in 48-hour ultrarunners. An AKI model of 90% accuracy and sensitivity was hypothesized to be possibly constructed.

This study aimed to build an AKI prediction model for ultrarunners during the prerace stage. We hypothesized that such a model could achieve at least 90% accuracy in AKI prediction. Volunteers who participated a 24-hour Ultramarathon Festival were recruited and their blood, urine, and body composition data were collected during prerace and postrace stages. SVM models were devised to compute the risk of acquiring AKI based on the prerace data of ultrarunners, and statistical analysis was performed to observe the physiological changes in ultrarunners. The proposed AKI prediction model may provide substantial AKI risk evaluation to ultrarunners before the start of a 24-hour ultramarathon.

## Methods

### Participants

This study was approved by the Joint Institutional Review Board (201309022), and all the recruited ultrarunners provided written consent forms to participate in the study. A total of 22 ultrarunners meeting the inclusion criteria (21 males and one female) volunteered to join the study. All the volunteers were ultrarunners competing in the ultramarathon held at the 2015 24-hour Ultramarathon Festival. The study’s exclusion criteria were as follows: (1) history of syncope of unknown origin; (2) chest pain of unknown origin; (3) difficulty breathing of unknown origin; (4) history of heart disease (including congenital heart disease, coronary heart disease, and heart failure); (5) any musculoskeletal injury that may affect physical performance; (6) history of renal dysfunction; (7) history of seizure; (8) reluctance to provide biochemical samples; and (9) unwillingness to follow the study procedures.

### Measures

Blood samples and body composition of each of the 22 participants were measured 1 h before and after the race. Body composition data were measured using Bioscan920II, and blood samples were collected using sterile techniques. Blood (20 mL) was drawn from the antecubital vein from each study participant 1 h before and immediately after the race. All specimens were refrigerated and transported to the laboratory within 4 h of sampling. Plasma samples were assayed on the Siemens Dimension RXL Max Integrated Chemistry System using reagents supplied by the manufacturer. Analysis was performed on the day of the race using the same calibration. Troponin I was analyzed using a high-sensitivity cTnI assay (Siemens Healthcare Diagnostics, Germany). Creatine kinase (CK), CK-muscle/brain MB (CK-MB) isoenzyme, electrolytes, renal function indices, lipid metabolism indices, and myoglobin (MYO) were analyzed using the Siemens Dimension RxL Max Integrated Chemistry System (Siemens Dimension RxL, Germany), with reagents supplied by the manufacturer. Urine was also collected to analyze BUN, creatinine, MYO (Miditron M, ROCHE), and electrolytes (Medica Corporation, EasyLyte®).

### Design and Procedures

All ultrarunners began the race at 3:00 P.M. on February 13, 2015, and ended at 3:00 P.M. on February 14, 2015. During the race, ultrarunners consistently ran around the 668 m path. They were permitted to rest, consume water, and take in food freely during the race. All the ultrarunners were required to complete an application form for demographic data and information on the medical and training history at the pre-race stage. Prerace height and body mass were measured by study personnel. Blood samples and body composition of each of the 22 participants were measured 1 h before and after the race, respectively.

The definition of AKI in this study was based on the serum creatinine levels obtained from the Acute Kidney Injury Network criteria (Mehta et al., 2007). According to the criteria (Mehta et al., 2007), AKI has three stages. For stage 1 AKI, the postrace plasma creatinine levels of ultrarunners are 1.5–2 times the prerace levels or they exhibit an increase of 0.3 mg/dL compared with the prerace levels. For stage 2 AKI, the postrace plasma creatinine levels of ultrarunners are 2–3 times the prerace levels. The ultrarunners are classified under stage 3 AKI if their postrace plasma creatinine levels are more than three times their prerace levels, with an acute increase larger than or equal to 0.5 mg/dL.

### Statistical Analyses

The Friedman test was selected to determine changes in blood, urine, and body composition data from prerace to postrace because of the sample size (Hsu et al., 2020; Kao et al., 2015).

The blood data included in this study consisted of blood urea nitrogen (BUN, mmol/L), creatinine (mg/dL), sodium (Na, mmol/L), potassium (K, mmol/L), glomerular filtration rate (GFR, mL/min), high-density lipoproteins (HDLs, mg/dL), triglyceride (TG, mg/dL), low-density lipoproteins (LDLs, mg/dL), cholesterol (CHOL, mg/dL), CK (U/L), CK-MB (U/L), MYO (ng/dL), and troponin (TROP T, μg/L).

Urine data comprised five indices: BUN (mmol/L), creatinine (mg/dL), Na (mmol/L), K (mmol/L), and MYO (ng/dL).

Body composition data included the measurements obtained using Bioscan920II. A total of 29 items were included in the body composition category: the basal metabolic rate (BMR) (kcal), fat-free mass (FFMKG, kg), the fat-free mass ratio (FFM, %), fat (FATKG, kg), the fat ratio (FAT, %), total body water volume (TBW, L), the total body water volume ratio (TBW100, %), extracellular water volume (ECW, L), the extracellular water volume ratio (ECW100) (%), intracellular water volume (ICW) (L), the intracellular water volume ratio (ICW100, %), the ECW to ICW ratio (ECWICW), body cell mass (BCM, kg), extracellular mass (ECM, kg), creatinine clearance (CCR, mL/min), GFR (mL/min), protein mass (kg), mineral mass (kg), muscle mass (kg), total body K (TBK, g), total body calcium mass (TBCa, g), glycogen mass (g), dry weight (kg), extracellular solids (ECS, L), extracellular fluid (ECF, L), plasma fluid (PF, L), interstitial fluid extra vascular (InterstF, L), body volume (L), and body density mass (kg).

### AKI Risk Evaluation Model Construction

In this study, four AKI prediction models were proposed to achieve AKI risk evaluation during the prerace stage. Three of the four models were built on the basis of blood, urine, and body composition data separately. The last model was constructed based on the combination of all three collected datasets. All four models were built using the SVM algorithm developed by Hsu et al. (2020), with modifications in the input and output of the algorithm. Moreover, a similar recursive SVM algorithm has been applied by Zhang et al. (2006) in bioinformatics. Every computational task was run on MATLAB 2019a.

**Figure 1:**
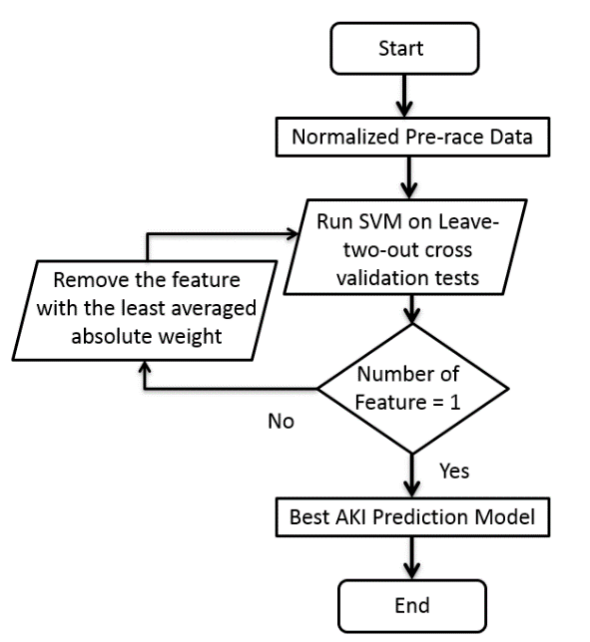
Flowchart of the construction of the AKI risk evaluation model.

The SVM model (Figure 1) treated the prerace data and the AKI indicator of ultrarunners as input and generated the best AKI risk evaluation model as output. With the exclusion of the AKI indicator, the input of the SVM model was the normalized prerace blood, urine, and body composition data of ultrarunners. The normalization utilized in this study transformed the data into zero means and unit variances [standard deviation (STD) = 1] by reducing the mean and scaling the data with the calculated STD. Meanwhile, the AKI indicator was a binary vector composed of zeroes and ones.

After the data were normalized, the linear kernel SVM model was ran on the 11-fold cross-validation test at each iteration to determine the contributing factors of AKI prediction. For each cross-validation test, an SVM model with 20 samples was built, and the two left-out samples were used to determine the performance of the built SVM model. The 11-fold cross-validation test was chosen based on the rule of leaving 10% data out for validation in the dataset with a relatively small sample size. In each iteration, the weight of each feature, accuracy, sensitivity, and specificity of AKI prediction were recorded. Each feature was one normalized input component (e.g., normalized creatinine from the blood sample), and the weight of each feature corresponded to the quantified contribution of that feature to evaluating the risk of acquiring AKI. Accuracy, sensitivity, and specificity were defined using formulas applied in the Hsu et al.’s (2020) study as follows:


$$\begin{array}{rl} \text{ accuracy } & =\frac{\hat{Y}}{Y}\times 100\text{\%}\\ \text{ sensitivity } & =\frac{{\hat{Y}}_{AKI}}{Y_{AKI}}\times 100\text{\%}\\ \text{ specificity }= & \frac{{\hat{Y}}_{noAKI}}{Y_{noAKI}}\times 100\text{\%} \end{array}$$

where Y is the label of the data, and Ŷ is the predicted label of the model. The subscripts in AKI were the data subset of those acquiring AKI, while “noAKI” concerned the subset of subjects without AKI. Once the execution of the 11-fold cross-validation was completed in one iteration, the SVM model verified whether the number of features was equal to one. Subsequently, when it was true, the best AKI risk evaluation model was returned, which was equivalent to the model with the highest averaged accuracy (averaged across the results of the cross-validation tests). Otherwise, the SVM model removed the feature with the minimum averaged absolute weight (least contribution to AKI prediction), and the data with the removed feature entered the next iteration.

### Quantification of AKI Risk Evaluation Models’ Performance

Performance of the four proposed models was quantified to determine the best 24-hour ultramarathon AKI prediction model. The average accuracy, sensitivity, and specificity of each best model (the output of the SVM model) were selected as performance quantification metrics. In addition, the overall accuracy, sensitivity, and specificity of the entire dataset were computed, and an 11-fold cross-validation test was performed with the best AKI prediction model, considering that the evaluation conducted on the entire dataset exhibited the goodness of the model, and the results of the cross-validation test could represent the model’s reliability.

## Results

### Demographics and AKI Outcomes

The demographics and AKI outcomes of the recruited participants are shown in Table 1. In this study, 22 subjects, with 21 men and one woman, were recruited. The average age of the recruited ultrarunners was 44 years, with the age spanning from 28 to 67 years. Ten out of 22 participants (45%) were diagnosed with stage 1 AKI immediately after the race, while the remaining 12 participants had no AKI diagnosis. None of the ultrarunners were classified as stages 2 and 3 AKI.

**Table 1:** Demographics and Acute Kidney Injury Outcomes in 24-Hour Ultramarathon Runners (n = 22).

Variables	Outcome
Age	44 (22-58) years
Gender	21 males, 1 female
Body Height	171 ± 4.08 cm
Body Mass	67.9 ± 10.5 kg
Body Mass Index	23.1 ± 3.10 kg/m^2^
AKI Stage 0	12 (55%)
AKI Stage 1	10 (45%)

### Analyses of Biochemical Data

Tables 2–4 present the Friedman test results of the ultrarunners’ blood, urine, and body composition data between 1 hour before and after the race. Blood levels of BUN, creatinine, CK, CKMB, TROP T, MYO, and HDL were significantly increased after the race. On the contrary, blood levels of GFR, TG, LDL, and CHOL were significantly decreased. Urine BUN, creatinine, K, and MYO levels were significantly increased. As for body mass data, the levels of muscle, dry weight, and body density were found to be significantly reduced.

**Table 2:** Biochemical Data of Blood at Prerace and Immediately After the Race in 24-Hour Ultramarathon Runners (n = 22) [* denotes p < 0.05].

Component	Pre-race Average (Range)	Immediately After Race Average (Range)	*p* Friedman Test
BUN (mmol/L)	14.6 (10–23)	25.5 (13–41)	<<0.001*
Creatinine (mg/dL)	0.77 (0.6–1.0)	1.02 (0.8–1.3)	<<0.001*
GFR (mL/min)	123.8 (92–188)	86.7 (60–130)	<<0.001*
Na (mmol/L)	140.3 (137–144)	140.7 (134–150)	0.49
K (mmol/L)	4.28 (3.7–4.9)	4.38 (3.8–5)	0.49
CK (U/L)	164 (72–397)	5697 (701–14391)	<<0.001*
CKMB (U/L)	22.5 (12.7–39.3)	195 (32.1–605)	<<0.001*
TROP T (ug/L)	0.0048 (0.0030–0.0170)	0.011 (0.0030–0.030)	<<0.001*
MYO (ng/dL)	26.3 (10.6–57.4)	1479 (91.1–4874)	<<0.001*
HDL (mg/dL)	66.6 (46–91)	76.3 (56–102)	<0.001*
TG (mg/dL)	129 (44–390)	58.6 (28–114)	<<0.001*
LDL (mg/dL)	123 (64 – 171)	92.7 (10–151)	<<0.001*
CHOL (mg/dL)	223 (165–321)	191 (139–255)	<0.001*

**Table 3:** Biochemical Data of Urine at Prerace and Immediately after the Race in 24-Hour Ultramarathon Runners (n = 22) [* denotes p < 0.05].

Component	Pre-race Average (Range)	Immediately After Race Average (Range)	*p* Friedman Test
BUN (mmol/L)	626 (152–1831)	1510 (496–2065)	<0.001*
Creatinine (mg/dL)	84.5 (18.3–228)	192 (37–297)	<0.001*
Na (mmol/L)	112 (34–260)	65.8 (15–140)	0.01*
K (mmol/L)	38.5 (9.6–90)	78.9 (26.4–124.5)	<0.001
MYO (ng/dL)	1.05 (1–2.2)	147 (1–1600)	0.002

**Table 4:** Biochemical Data of Blood at Prerace and Immediately after the Race in 24-Hour Ultramarathon Runners (n = 22) [* denotes p < 0.05].

Component	Pre-race Average (Range)	Immediately After Race Average (Range)	*p* Friedman Test
BMR (kcal)	1739 (1475–2014)	1725 (1470–2132)	0.67
FFMKG (kg)	55.8 (45.0–67.4)	55.1 (44.7–69)	0.67
FFM (%)	82.8 (62.0–95.1)	83.4 (66.4–92.9)	0.39
FATKG (kg)	12.2 (3.11–38.8)	11.5 (3.60–33.9)	0.28
FAT (%)	17.2 (4.94–38.0)	16.6 (7.14–33.6)	0.39
TBW (L)	43.1 (31.8–66.6)	40.4 (28.7–57.9)	0.39
TBW100 (%)	63.9 (47.4–90.0)	61.2 (46.2–80.1)	1
ECW (L)	18.3 (14.9–30.1)	17.9 (15.2–24.1)	1
ECW100 (%)	42.9 (35.8–48.4)	44.5 (41.6–62.6)	0.09
ICW (L)	24.7 (16.4–36.9)	24.7 (16.4–36.9)	0.20
ICW100 (%)	57.1 (51.6–64.2)	55.4 (37.4–58.4)	0.09
ECWICW	0.75 (0.56–0.94)	0.82 (0.71–1.68)	0.09
BCM (kg)	30.8 (22.9–38.9)	29.3 (21.4–37.5)	0.09
ECM (kg)	25.0 (21.8–28.9)	25.8 (21.7–32.9)	0.83
CCR (mL/min)	318 (4.80–978)	486 (0.3–995)	0.39
GFR (mL/min)	83.2 (43.2–108)	78.7 (42.6–104)	0.09
PROTEIN (kg)	9.40 (0.56–14.1)	10.8 (4.81–19.0)	0.67
MINERAL (kg)	3.32 (0.20–4.93)	3.82 (1.69–6.66)	0.67
MUSCLE (kg)	27.2 (19.4–32.6)	26.8 (19.2–34.4)	0.02*
TBK (g)	147 (102–186)	140 (100–179)	0.09
TBCa (g)	1185 (857–1467)	1135 (848–1420)	0.09
GLYCOGEN (g)	507 (409–612)	500 (405–627)	0.67
DRY WEIGHT (kg)	66.3 (49.3–99.8)	64.5 (48.3–98.5)	<0.001*
ECS (L)	6.01 (4.35–7.45)	5.76 (4.31–7.20)	0.09
ECF (L)	19.4 (15.8–31.9)	19.0 (16.1–25.5)	1
PF (L)	3.88 (3.15–6.38)	3.79 (3.22–5.10)	1
InterstF (L)	13.6 (11.0–22.3)	13.3 (11.3–17.9)	1
Body Volume (L)	64.2 (47.8–100.6)	62.8 (46.5–98.6)	<0.001*
Body Density (kg)	1.06 (1.01–1.09)	1.06 (1.02–1.08)	0.39

### Outcomes of SVM Models

The selected features of each SVM model are provided in Table 5, and all the features are listed in the decreasing order of contribution in AKI prediction. Creatinine, TG, and GFR were the components of the blood AKI prerace risk evaluation model. Creatinine and BUN were the selected features in the urine AKI prerace risk evaluation model. BCM, ECM, and BMR were selected from the body composition data to evaluate the risk of acquiring AKI during the prerace stage. In the decreasing order of contribution, creatinine from the blood data, InterstF, ECW, TG, and BUN from the blood data; ECM, BMR, and K from the urine data; PF and K from the blood data; and creatinine from the urine data were used to determine the risk of AKI during the prerace stage. In the mixed model, features from the blood, urine, and body mass data were selected.

**Table 5:** Features and their weights of the Four Proposed AKI Pre-race Risk Evaluation Models.

Model	Features
Blood	CREA+, TG-, GFR-
Urine	CREA+, BUN-
Bodymass	BCM-, ECW+, BMR-
Mixed	blood Creatinine+, InterstF-, ECF-, TG+, blood BUN-, ECM-, BMR+, urine K-, Plasma F+, blood K+, urine Creatinine-

Features are listed in decreasing order of the contribution of AKI risk evaluation. + / -: the larger the feature, the lower / higher the risk of getting AKI

In addition to the ranking of each feature’s contribution, Table 5 presents the relationship between the level of the feature and its risk of exposure to AKI. In the blood AKI prediction model, the higher the levels of creatinine and GFR were, the lower the risk of 24-hour ultrarunners acquiring AKI. Moreover, the higher the TG levels were, the higher the risk of exposure to AKI. In the urine AKI prediction model, 24-hour ultrarunners with high creatinine and low BUN levels tended to acquire AKI. Low BCM, low BMR, and high ECM levels increased the risk of 24-hour ultrarunners to acquire AKI in the body composition model. In the mixed model, ultrarunners were prone to acquire AKI if they had high levels of creatine, TG, BMR, PF, and K in the blood and low levels of InterstF, ECF, and BUN in the blood; ECM and K in the urine; and urine creatinine.

### AKI Risk Evaluation Models during Prerace Stage

Table 6 exhibits the training and cross-validation results of the four proposed AKI risk evaluation models. On average, the mixed model exhibited the best performance. Training accuracy, sensitivity, and specificity of the mixed model were 96%, 90%, and 100%, respectively. Moreover, the mixed model’s cross-validation accuracy, sensitivity, and specificity were 90%, 83%, and 95%, respectively. Referring to the performance of the blood, urine, and body mass models, their training accuracy, sensitivity, and specificity were within the ranges of 55−80%, 10−60%, and 92−100%, respectively, and their cross-validation accuracy, sensitivity, and specificity were within the ranges of 45−70%, 0−60%, and 75−96%, respectively.

**Table 6:** Training and Cross Validation Results of the Four AKI Pre-race Risk Evaluation Models.

Model	Training			Cross Validation		
	Accuracy	Sensitivity	Specificity	Accuracy	Sensitivity	Specificity
Blood	73%	50%	92%	70%	45%	90%
Urine	77%	60%	92%	70%	56%	76%
Bodymass	59%	10%	100%	47%	0%	96%
Mixed	96%	90%	100%	90%	83%	95%

## Discussion

### First ML-based AKI Risk Evaluation Model of 24-hour Ultramarathon Runners during the Prerace Stage

This study, to the best of the authors’ knowledge, was the first to predict acquiring AKI among 24-hour ultrarunners before the start of the race. The best model reached 96% accuracy in training and 90% in cross-validation tests. Furthermore, 90% sensitivity and 100% specificity were achieved. The best model comprised a mixture of blood, urine, and body composition data from 24-hour ultrarunners. The proposed model could prevent ultrarunners from acquiring AKI at the prerace stage of a 24-hour ultramarathon.

The features of the best AKI risk evaluation model were creatinine, BUN, TG, and K from the blood data; K and creatinine from the urine data; and InterstF, ECF, ECM, BMR, and PF from the body composition data. In addition, these 11 components indicated that certain levels of biochemicals, lipoproteins, and ions, together with additional body composition information, could infer the chance of an ultrarunner being exposed to AKI in a 24-hour ultramarathon.

A closer look at the features of the best model revealed three main indicators included in the AKI risk evaluation model for 24-hour ultramarathon runners: creatinine levels from blood and urine, the basic metabolic rate, and balance of body fluid and electrolytes. A significant correlation between muscle mass and creatinine has been reported by Baxmann et al. (2008). An Elevated BMR has been observed in individuals undergoing long-term physical activity (Speakman and Selman, 2003). According to Rehrer’s (2001) research, balance of body fluid and electrolytes could influence performance of athletes in ultra-endurance sports. On the basis of the aforementioned publications, the outcomes of the AKI risk evaluation model are reasonable. Furthermore, ultrarunners participating in 24-hour ultramarathons are suggested to build up muscle mass and train themselves in ultra-endurance sports.

By contrast, models which utilized a single type of data exhibited varying performance and did not surpass that of the mixed model. Among blood, urine, and body composition, the urine model generally achieved the best performance.

### Biochemical and Physiological Changes

Statistical findings of biochemical and physiological changes were compared with those of other related studies in traditional marathons and ultramarathons. The current study provided consistent responses as other related research works. The similar findings include the decrease in fat content, elevated glycogen, the breakdown of muscle (measured from BioScan920), and variation in ions in urine and blood (Kao et al., 2015; Wu et al., 2004). The consistency of the obtained results further strengthened the validity of the data collected in the present study.

## Conclusions

In summary, we acknowledge that there are some limitations to our study. The relatively small sample size and the observational design limit the overall strength of the conclusions. Due to the limited number of participants available in this study, a more detailed biochemical analysis with a much larger number of samples would be required to elucidate the molecular mechanisms involved. On the other hand, ultra-marathons are rather exclusive events with far fewer participants compared to regular marathon races, and we believe that our study, despite a small sample size, is of importance. In our study, an AKI prediction model achieving 96% accuracy was constructed, which supports the stated hypothesis of building an AKI prediction model accomplishing at least 90% accuracy. A promising model for evaluating the risk of acquiring AKI among 24-hour ultramarathon runners at the prerace stage was demonstrated. Such an AKI prediction model considered blood, urine, and body composition data as input and output in AKI prediction, and it successfully achieved accuracy of 96%. Considering the components of the proposed model, ultrarunners with relatively high muscle mass and low-fat mass have a lower risk of acquiring AKI (Hsu et al., 2020). Therefore, ultrarunners are suggested to have high a high % of FFM, but a low body mass, and regular ultra-endurance sports training. Furthermore, the model could potentially extend translational applications to other extreme sports for injury prevention and risk evaluation for diagnosis.
